# Twenty Years of European Hair Research Society

**DOI:** 10.4103/0974-7753.51916

**Published:** 2009

**Authors:** Ralph M Trüeb

**Affiliations:** EHRS President, Department of Dermatology, University Hospital of Zurich, Gloriastr. 31, 8091 Zurich, Switzerland. E-mail: ralph.trueeb@usz.ch


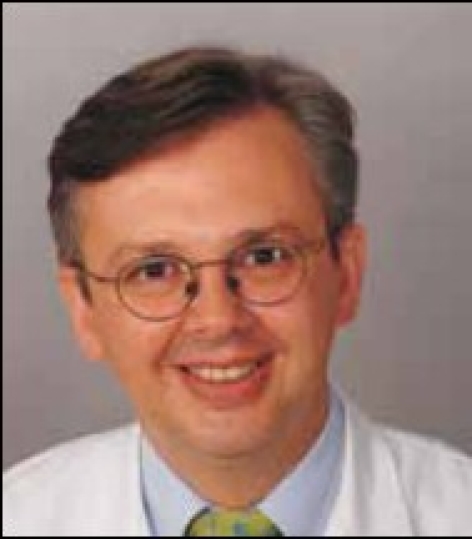


The idea of establishing the first independent, nonprofit organization for hair research was launched on April 1989 by a small group of enthusiastic hair scientists and was soon followed by its formal constitution on the occasion of the first and founding meeting of the European Hair Research Society (EHRS) on November 1989 in Brussels, Belgium.

Since then the EHRS has organized annual meetings in different European cities dedicated to the presentation of new research findings, exchange of information, education of its members, and the advancement of science, or has co-organized international scientific meetings with the North American Hair Research Society, the Society for Hair Science Research (Japan), and the Australasian Hair and Wool Research Society. Since then the world of hair research has also expanded to include new sister societies, including the Korean Hair Research Society and the Indian Hair Research Society. Since its constitution in 1989, the EHRS has continued to grow in numbers and influence that reach far beyond the geographic boundaries of the European continent. Today, the EHRS embraces an international membership of scientists, clinicians, and professionals working at academic institutions, in private practice, and in the industry, and also encourages the scientific careers of young investigators and entertains a close cooperation with its international sister societies across the globe.

It is my pleasure to present herein the abstracts of the 2009 EHRS meeting, July 2–4 in Graz, Austria. The Congress President, Daisy Kopera, the Organizing and the Scientific Boards have committed themselves to offer the latest highlights in hair research and clinics. It is a special occasion for celebrating 20 years of EHRS, and at the same time the first issue of the *International Journal of Trichology*. I wish both many more years to come!

